# Trace-level detection of sulfonamide antibiotics using quaternary ammonium polymeric ionic liquid-based effervescence-enhanced dispersive solid-phase extraction followed by LC-DAD analysis in environmental waters†

**DOI:** 10.1039/d2ra02488h

**Published:** 2022-10-19

**Authors:** Sai Ma, Ming Gao, Su Ma, Jun Wang, Yue Sun, Hanyu Wang, Huili Wang, Xuedong Wang

**Affiliations:** School of Environmental Science and Engineering, Suzhou University of Science and Technology Suzhou 215009 China whuili@163.com zjuwxd@163.com; School of Chemistry and Life Sciences, Suzhou University of Science and Technology Suzhou 215009 China

## Abstract

Conventional ionic liquids possess several disadvantages, such as high viscosity, difficult sampling/retrieval, and great loss in aqueous solution, limiting their wide applications in the pretreatment field. To solve these drawbacks, we synthesized a quaternary ammonium polymeric ionic liquid (PIL) and pressed it into an effervescent tablet for developing an effervescence-enhanced dispersive solid-phase extraction method (QAP-EDSE). The pressed effervescent tablet was composed of PIL as an extractant, tartaric acid as an acidic source, NaHCO_3_ as an alkaline source, and water-soluble starch as a filler, respectively. Under the CO_2_-driven dispersion, the QAP-EDSE method integrated rapid enrichment, extraction, and dispersion into one synchronous step. Employing the one-factor-at-a-time approach, several important variables were optimized as follows: 200 mg of P[VBTHEA]Cl as sorbent, 400 μL of acetone as elution solvent, 5 min of elution, solution pH 9.0, and 1 : 1.25 molar ratio of alkaline to acidic sources. Combining LC-DAD analysis, this proposed approach offered the limits of detection as low as 0.11–0.31 μg L^−1^ and satisfactory recoveries of 81.40–102.62% for five sulfonamides (SAs) in environmental waters. The lower relative standard deviations (1.9–6.7%) evidenced the higher intraday and interday experimental precision by this method. Overall, the newly developed method is environmentally benign, time-saving, and easy to operate with low detection limit and high recovery and thus shows excellent prospects in the trace-level detection of SAs in environmental waters.

## Abstract 2

An effervescent tablet-assisted dispersive solid-phase extraction based on the utilization of quaternary ammonium poly ionic liquids (PIL) was proposed for the concentration/extraction of sulfonamides (SAs) in river and lake water samples.

## Graphical abstract

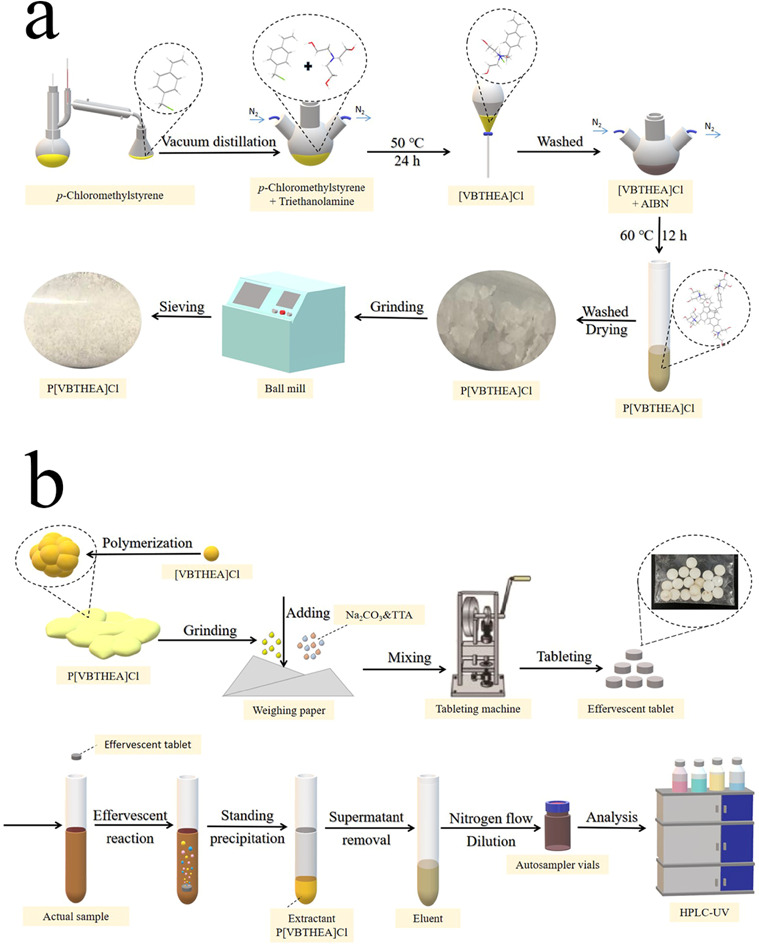

## 1. Introduction

Sulfonamides antibiotics (SAs), belonging to amphoteric antimicrobial compounds, are extensively used as bactericidal and bacteriostatic agents in human medicine and livestock industry.^1^ SA metabolism requires long time in the human body, and thus a large amount of drug prototypes are excreted in the feces and urine.^2^ They can eventually enter environmental water bodies with runoff from agricultural fields and as effluents from wastewater treatment plants.^3^ Among commonly used SAs, five species are frequently detected at the levels of 10–500 ng L^−1^ in rivers, lakes, and groundwater,^4^ which include sulfamethoxydiazine (SMD), sulfadimethoxine (SDM), sulfathiazole (ST), sulfamethoxazole (SMZ), and sulfadiazine (SD). Their indiscriminate use or prolonged contact with residues may lead to antibiotic resistance in both veterinary and human applications. On the other hand, they are found to be carcinogenic and have relatively long half-lives.^5^ Therefore, the widespread use of SAs risk the contamination of food and their metabolites endanger the health of consumers. Notably, because SAs are often used in animal feed, their residues are frequently detected in some agricultural products and livestock wastewater.^6,7^ For example, Jansomboon's group reported that the highest concentrations of SMD, SD, ST, and SDM spanned the range of 6.23–245.91 ng g^−1^ in imported *Pangasius* catfish products in Thailand, which were close to or higher than the European Union (EU) standard.^8^ It is well known that large doses of SAs over a short period can cause acute poisoning, and chronic poisoning can be caused by small doses of SAs over a long period, including allergies and hematological, immune, urinary, and neurological disorders,^2^ as well as potential carcinogenicity.^9^ In addition, many fungi will develop resistance to SAs when they remain in the body for a long time.^10^ As a consequence, it is crucial to develop simple, sensitive, robust, and ecofriendly methodologies for the determination of SAs in environmental waters.

Currently, various pretreatment methods for SAs detection have been developed, including liquid-phase microextraction (LPME),^11^ solid-phase extraction (SPE),^12^ and solid-phase microextraction (SPME).^13^ Although these approaches have satisfactory extraction efficiencies for SAs, they have many distinct shortcomings, such as the use of toxic solvent, time-consuming, tedious operation, and high cost.^14^ Dispersive solid-phase extraction (d-SPE) is a modification of SPE, in which the target analytes are extracted by adding the sorbent directly to the sample solution.^15^ After dispersion, the contact surface between the analyte and the sorbent is greatly enhanced, which effectively shortens the time for mass transfer and extraction equilibrium. Therefore, as compared to conventional SPE, d-SPE requires only mg-level sorbent and can complete the entire extraction process in a shorter time.

To realize rapid dispersion, adsorption, and elution of analytes, sorbents with large specific surface area, high adsorption efficiency, and good dispersive ability are required in the d-SPE techniques. Compared to inorganic sorbent materials such as carbon,^16^ silica,^17^and metal–organic frameworks (MOFs),^18^ organic materials have a wider variety of functional groups, which may improve the extraction efficiency and sorbent selectivity.^19^ Among organic materials, ionic liquids (ILs) are deemed as “green” extraction media and have designable structures, which provide multiple interactions with target molecules, including hydrophobic/hydrophilic interactions, hydrogen bonding, π–π conjugation, ion-exchange, and electrostatic attraction or repulsion.^20^ However, the applications of ILs in the pretreatment methods still have some distinct limitations due to high viscosity, difficult retrieval, and low utilization efficiency.^21^ In comparison, polymeric ionic liquids (PILs) possess the advantages of both ILs and polymers, and thus have good ionic and conductive properties,^22^ which expand their applications as the fit-for-purpose and functional ILs. PILs have higher thermal stability, better durability, improved mechanical robustness, and longer service life compared to ordinary ILs. Moreover, they possess similar characteristics as that of regular polymers, including processability, plasticity, and spatial controllability, and thus can be used as promising alternatives in several extraction methods.^23^ So far, PILs have been extensively employed in gas adsorption and separation, especially in CO_2_ adsorption, which solve the bottleneck problem of ILs in high viscosity and low-adsorption selectivity.^24^ Although there have been many reports on the utilization of PILs as catalysts and adsorbents, few reports are concerned with their applications as extractants the pretreatment procedures.

The dispersion of sorbents in the d-SPE methods is usually assisted by the introduction of an external energy source, such as sonication, stirring, vortexing, or microwave;^25^ thus, it usually requires a long reaction time and specialized equipment. Effervescence-enhanced extraction is based on a simple acid–base reaction to generate CO_2_ bubbles to promote the dispersion of extraction solvents or/and adsorbent particles.^26^ The generated bubbles enlarge the contact surface area between the extractant and the sample solution and promote mass transfer, thereby enhancing extraction efficiency. In this investigation, effervescent tablets were introduced into the d-SPE procedures, which eliminated the need of physical energy and special equipment to disperse the sorbents, thereby simplifying the operational process and shortening the extraction time.^27^

Using the above information, we herein synthesized a quaternary ammonium PIL (P[VBTHEA]Cl) and employed it in an effervescence-enhanced dispersive solid-phase extraction method, hereafter abbreviated as QAP-EDSE. The application of solid P[VBTHEA]Cl not only solves the disadvantages of conventional ILs (high viscosity, difficult retrieval, and great loss in aqueous phase) but also realizes the specific adsorption/extraction of SAs due to rich amino groups in this PIL. In the QAP-EDSE method, P[VBTHEA]Cl was used as an adsorbent/extractant in aqueous solution, and its rapid dispersion was achieved with the aid of vigorous CO_2_ bubbles from an acid–base effervescent reaction. Consequently, it integrated concentration, extraction, and quick dispersion into one synchronous step. Combined with LC-DAD detection, this proposed method provided satisfactory analytical indicators for trace-level SAs detection in environmental waters, and thus shows excellent prospects in the sample pretreatment field.

## 2. Materials and methods

### 2.1. Reagents and chemicals

Five SAs (SMD, SDM, ST, SMZ, and SD), each with purity > 99.0%, were purchased from China Chemical Standard Corporation (Beijing, China), and their molecular structures and physico-chemical characteristics are listed in ESI Table 1.† The analytical-grade chemicals were all obtained from Shanghai Tixiai Chemical Corporation (Shanghai, China): 4-vinylbenzyl chloride (4-VBC), bromethane, triethanolamine (TEA), 2,6-di-*tert*-butyl-4-methylphenol (DBMP), azobisisobutyronitrile (AIBN), sodium bicarbonate (NaHCO_3_), and tartaric acid (TTA). Chromatographic-grade acetone, methanol, ethyl alcohol, and acetonitrile were sourced from Aladdin (Shanghai, China).

### 2.2. Instrumentation

The morphology of (P[VBTHEA]Cl) was characterized by scanning electron microscopy (SEM, Sigma 300, Germany), and its corresponding FT-IR spectrum was measured with a scanning range of 400–4000 cm^−1^ by the KBr method in a Bruker Tensor II infrared spectrometer (Brook, Germany). X-ray photoelectron spectroscopy (XPS) was determined using a PHI Quantera Spectrometer with AlKα X-ray (*hν* = 1486.6 eV) radiation. The thermal stability of P[VBTHEA]Cl was detected by a SDT Q600 thermogravimetric analyzer (PerkinElmer, MA, USA). The Brunauer–Emmett–Teller (BET) surface area was measured by N_2_ adsorption–desorption at 77 K using an ASAP 2020 System (Quantachrome, USA). Zeta potential was detected by a zeta potential analyzer (Malvern, UK). The solution pH was measured by a Leici PHB-4 pH meter (Inesa Scientific Corporation, Shanghai, China). Ultrapure water (>18.2 MΩ) was generated with a Hangzhou Yongjieda purification system (Hangzhou, China). The nylon membrane filter (50 mm × 0.45 μm) was acquired from Tianjin Jinteng Chemical Corporation (Tianjin, China).

### 2.3. Detection of SAs by LC-DAD

The concentrations of five SAs were detected by a Shimadzu LC-20AT liquid chromatograph equipped with a diode-array detector (LC-DAD). Chromatographic separation was conducted on a Shim-Pack GIST C_18_ column (250 mm × 4.6 mm, 5 μm), which was operated under the following conditions: mobile phase, acetonitrile-water at 35% : 65% (v/v) for 15 min; flow rate, 0.8 mL min^−1^; and column temperature, 30 °C. The detection wavelength was set at 270 nm, and the injection volume was 10 μL.

### 2.4. Identification of SAs by LC-MS/MS

The identification and quantification of the five SAs in water samples were accomplished on an LC-MS/MS (AB Sciex API 4000, Los Angeles, CA, USA), which was equipped with an electrospray ionization (ESI) ionization probe. A CNW Athena C18-WP analytical column (2.1 mm × 100 mm, 3 μm particle size) was used for separations. The mobile phase consisted of methanol (v/v) (solvent A) and ultrapure water with 0.1% (v/v) formic acid (solvent B), and the optimized program was as follows: 0–7.0 min = 10–75% A, 7.0–7.2 min = 75–10% A, and 7.2–9.0 min = 10% A. At the same time, the flow rate was set at 0.25 mL min^−1^, column temperature was set at 30 °C, and the injection volume was 10 μL. To enhance the sensitivity and selectivity for the detection of the target SAs, MS analysis was performed using multiple reaction monitoring (MRM) with positive ESI mode. The mass parameters including ionization mode, parent ion, product ion, collision energy, and cone voltage for each analyte were optimized and are shown in ESI Table 2.† The parameters of the mass spectrometer were as follows: the capillary voltage and temperature were 3500 V and 350 °C, respectively, argon was used as the collision gas and the sheath gas and auxiliary gas pressure were 20 MPa and 5 MPa, respectively.

### 2.5. Fabrication of the [VBTHEA]Cl

**Fig. 1:**
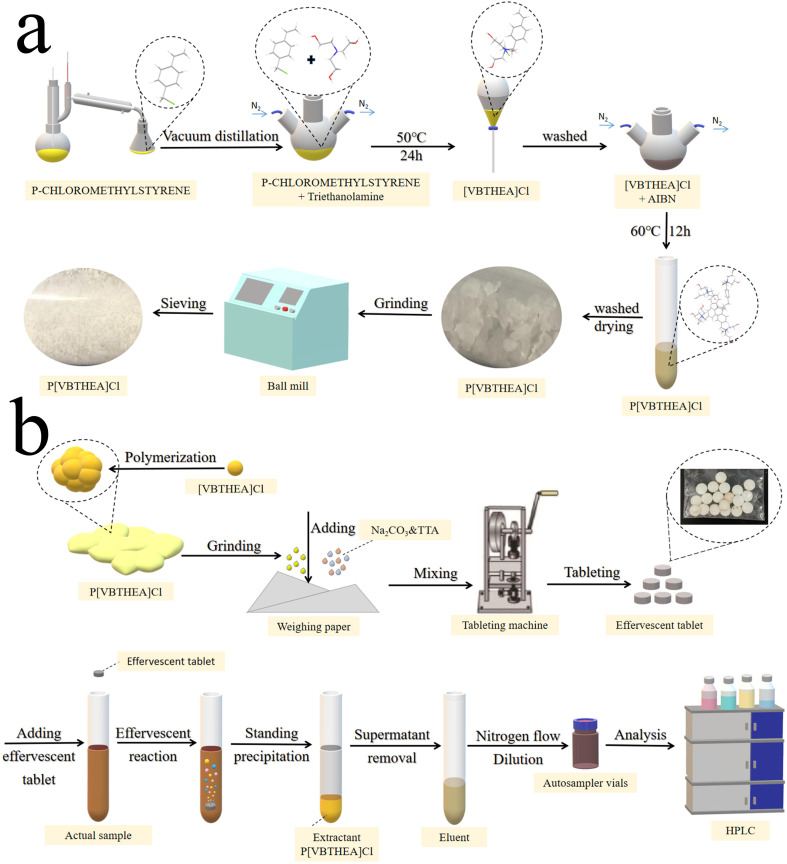
The schematic procedures for the synthesis of P[VBTHEA]Cl (a), preparation of effervescent tablets and QAP-EDSE operations (b).

Firstly, the main reactant 4-VBC was purified by distillation in a vacuum distiller to collect its 80–90 °C fractions to remove polymerization inhibitors and other impurities. Then, 4-VBC (7.65 g, 0.05 mol), TEA (7.46 g, 0.05 mol), anhydrous ethanol (20 mL), and DBMP (0.17 g) were introduced into a three-necked round-bottom flask and heated at 50 °C under a gentle N_2_ flow (Fig. 1a and ESI 1a†). The refluxing reaction of the mixed solution was kept for 24 h. The solution viscosity increased quickly and became larger. After the reaction, a yellow viscous liquid was acquired (ESI Fig. 2a–c†). Subsequently, the resultant liquid was washed with dichloromethane three times to remove the unreacted chemicals and polymerized inhibitors, and dried under vacuum for 24 h (Fig. 1a). The yield of [VBTHEA]Cl was 13.78 g with the yield rate of 91.2%.

### 2.6. Synthesis of P[VBTHEA]Cl

P[VBTHEA]Cl was synthesized based on free radical polymerization using AIBN as an initiator. Briefly, [VBTHEA]Cl (5.0 g), AIBN (0.06 g), and anhydrous ethanol (20 mL) were added to a 100 mL three-necked flask and heated at 60 °C under N_2_ atmosphere. With continuous reaction, we first observed the formation of yellowish white flocculent chemical, which was gradually accumulated into blocks (ESI Fig. 2d–f†). The resulting block solid was collected, ground, washed with dichloromethane, and dried under vacuum. The yield of P[VBTHEA]Cl was 4.48 g with a yield rate of 89.6%. The schematic diagram of the overall synthetic routes for P[VBTHEA]Cl is elaborated in ESI Fig. 1b†.

### 2.7. Preparation of the P[VBTHEA]Cl-based effervescent tablets

After optimizing the effervescent precursors, the mixture of NaHCO_3_ (2.0 g), tartaric acid (2.5 g), an approximate amount of P[VBTHEA]Cl, and water-soluble starch was carefully ground into a fine, homogeneous powder. An aliquot (∼0.80 g) of the homogeneous powder was compressed into an effervescent tablet (8 mm diameter × 2 mm thickness) using a T5 Single Punch Press (Chaoyi Machinery Factory, Shanghai, China). The as-fabricated tablets were stored in a sealed plastic bag in a desiccator. The detailed preparation process for effervescent tablets is exhibited in Fig. 1b.

### 2.8. Collection and preparation of water samples

Four water samples were collected from the Biyu River, Fuqian River, Shi Lake, and Taying Lake, Suzhou, China. Aliquots (30 mL) of the water samples were centrifuged at 5000 rpm for 10 min, and the supernatant was filtered through a 0.45 μm nylon membrane filter. The purified water samples were subjected to the subsequent effervescent reaction-enhanced microextraction procedures.

### 2.9. Procedures for the QAP-EDSE method

The overall operational procedures for the QAP-EDSE method are shown in Fig. 1b and ESI 3.† Firstly, the pretreated water sample (8.0 mL) was introduced into a 15 mL conical centrifuge tube (ESI Fig. 3a†). An effervescent tablet was placed into the solution, producing a continuous stream of CO_2_ bubbles due to an acid–base reaction (ESI Fig. 3b†). Obviously, the effervescent tablet was quickly disintegrated, and the PIL was diffused into the solution with the aid of vigorous dispersion from CO_2_ bubbles (ESI Fig. 3c–e†). After 2–3 min effervescent reaction, the resultant solution was kept still for 2 min (ESI Fig. 3f†). Subsequently, the supernatant was removed, and the sedimented PIL was eluted with 400 μL acetone, transferred to a 1.5 mL Eppendorf tube by a pipette, dried under gentle N_2_ flow, and quantitatively dissolved in 100 μL methanol. Finally, an aliquot (10 μL) of the above sample was collected and subjected to LC-DAD detection.

## 3. Results and discussion

### 3.1. Characterization of P[VBTHEA]Cl

#### 3.1.1. SEM and EDS analyses of P[VBTHEA]Cl

**Fig. 2:**
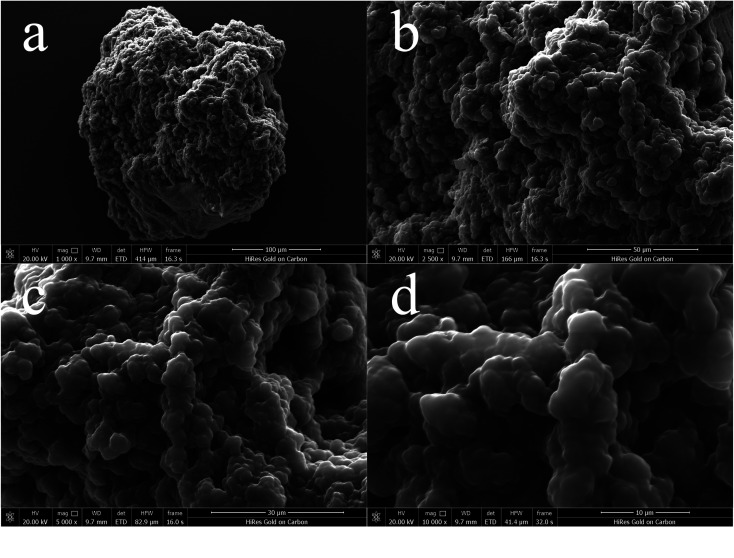
SEM images of P[VBTHEA]Cl at 1000 (a), 2500 (b), 5000 (c), and 10 000 (s) × magnification.

**Fig. 3:**
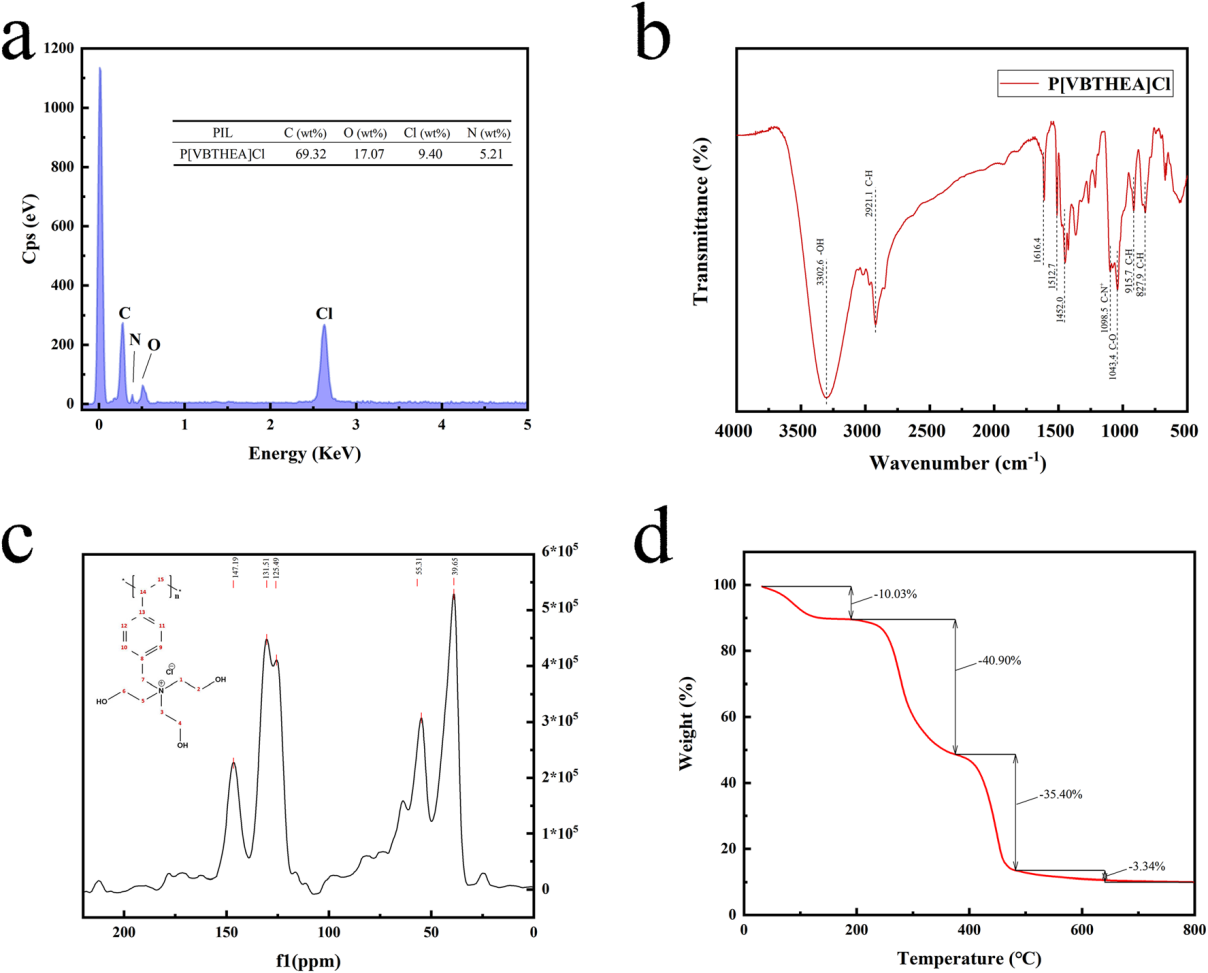
EDS mapping (a), FT-IR spectra (b), ^13^C NMR spectra, and TGA curve (d) of P[VBTHEA]Cl.

Fig. 2 displays four SEM images of P[VBTHEA]Cl at 1,000, 2,500, 5,000×, and 100 00× magnification. Obviously, this quaternary ammonium-based PIL presents irregular and asymmetric block structure with rough surface and no fixed crystal shape. From the overall appearance, P[VBTHEA]Cl is close to the shape of jade and piled up with different sizes of particles, demonstrating the formation of amorphous polymers.^28^ Elemental analysis by EDS mapping demonstrated the presence of C, O, Cl, and N in the as-synthesized nanocomposites, and the surface of P[VBTHEA]Cl consisted of 69.32% C (wt%), 17.07% O, 9.40% Cl, and 5.21% N (Fig. 3a), implying that the PIL P[VBTHEA]Cl was successfully polymerized with aid of the initiator AIBN.

#### 3.1.2. FT-IR and ^13^C NMR spectra of P[VBTHEA]Cl

As shown in Fig. 3b, the stretching-vibration peak at 3302.6 cm^−1^ is attributable to O–H, which indicates intramolecular or intermolecular hydrogen bonds,^29^ while the peak at 2921.1 cm^−1^ belongs to C–H on the methylene-CH_2_, and 1616.4 cm^−1^ and 1452.0 cm^−1^ are attributed to the characteristic absorption of the benzene skeleton. Comparatively, the peaks at 1098.5 cm^−1^ and 1043.4 cm^−1^ are attributable to C–N and C–O, while those at 915.7 cm^−1^ and 827.9 cm^−1^ are ascribed to the C–H bending vibration of benzene skeleton, respectively. In addition, the vibration intensity reflected the absorption value of the bond in the FT-IR spectrum, and obviously, the O–H vibration displays the strongest absorption among all the peaks.

The ^13^C NMR spectrum of P[VBTHEA]Cl is displayed in Fig. 3c. The peak at 147.19 ppm corresponds to two C atoms (positions 13 and 14) close to the link point between the vinyl group and the benzene ring. In contrast, the peak at 131.51 ppm is attributable to other two C atoms (positions 9 and 10) far from the link point, and that at 125.49 ppm is ascribed to two C atoms at positions 11 and 12. Similarly, the peak at 55.31 ppm corresponds to C (position 7) in the CH_2_–N^+^ chain, and the broad peak at 39.65 ppm is attributed to the C atoms of N^+^–CH_2_–CH_3_ at (positions 1, 2, 3, 4, 5, and 6).

#### 3.1.3. TGA, BET specific surface area (SSA), and pore size of P[VBTHEA]Cl

The TGA curve of P[VBTHEA]Cl was mainly divided into three stages (Fig. 3d). In the first stage, the weight loss was 10.03% below 200 °C, which resulted from the loss of water. For the second stage, additional weight loss of 40.90% occurred from 200 to 380 °C, possibly derived from the decomposition of N^+^–CH_2_–CH_2_–OH chain. In the third stage, ∼35.40% weight loss appeared, which came from the disintegration of the polymer framework.

**Fig. 4:**
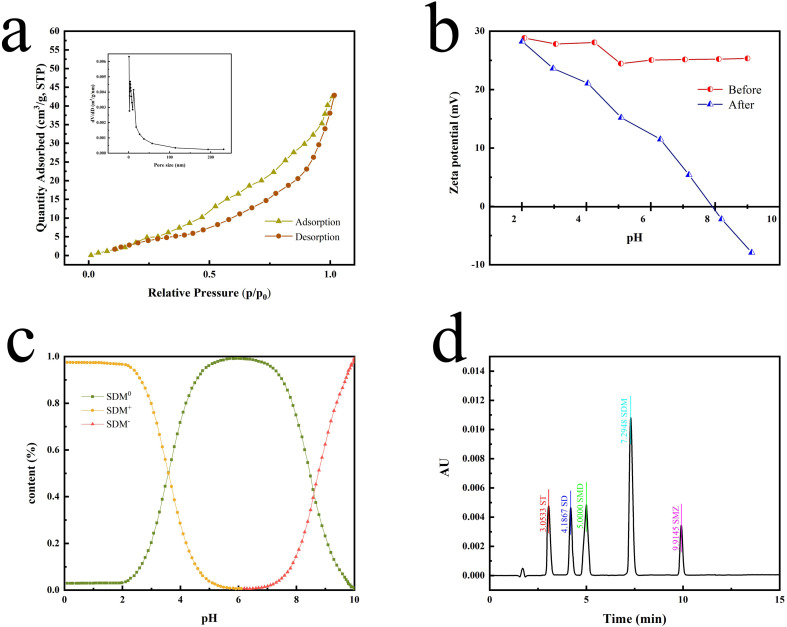
BET specific surface area (a), zeta potential (b), transition of SMD from the molecular state to the ionic state under varying solution pH (c), and chromatographic profiles of five SAs standards in ultrapure water (d).

According to the N_2_ adsorption–desorption curves (Fig. 4a), the BET SSA of P[VBTHEA]Cl was computed to be 22.7 m^2^ g^−1^, and its average pore size was 24.5 nm within the range of 2–50 nm for mesoporous materials (2–50 nm).^30^ In contrast to carbon-based nanomaterials, such as rGO, C_3_N_4_, and nanosheets, with SSAs of 100–600 m^2^ g^−1^,^30^ the smaller SSA of P[VBTHEA]Cl was not favorable to highly-efficient adsorption/extraction of SAs in the aqueous phase. Consequently, we conjectured that other action forces, such as H-bonding effect and electrical attraction, might play an important role in enhancing SAs adsorption/extraction.

#### 3.1.4. Zeta potential of P[VBTHEA]Cl

Fig. 4b elaborates the changes in the zeta potentials of P[BTQA]Cl at varying solution pH of 2.0–9.0. Evidently, they are positively charged on the surface of P[BTQA]Cl before microextraction. In stark contrast, the zeta potentials dramatically decrease with increasing solution pH after extraction, and they are positively charged across the pH range of 2.0–8.0 but negatively charged when the solution pH is > 8.0. As for all SA species, they have two dissociation constants (p*K*_a,1_ and p*K*_a,2_), which are in the range of 4.0–7.0.^31^ SAs present positive charges under strong acid conditions (pH < 5.0) but negative charges under strong base conditions (Fig. 4c). As a consequence, there is an electrostatic attraction between P[BTQA]Cl and the molecules of SAs under alkaline conditions, whereas a repulsive force under strong acidic conditions.

### 3.2. Optimization of the synthetic conditions for PIL

In the synthetic process, the spiked amount of anhydrous ethanol and initiator AIBN had significant effects on the physicochemical properties of P[VBTHEA]Cl. When the amount of anhydrous ethanol was too little (<15 mL), the synthesized [VBTHEA]Cl is highly viscous and even sticky (ESI Fig. 2c†), thereby producing too hard PIL and low yield. On the contrary (ethanol volume > 25 mL), [VBTHEA]Cl is too dilute to be favorable for the subsequent polymerization reaction with AIBN (ESI Fig. 2a†). Similarly, when too little and large amount of AIBN was added, the resulting PIL offered a low yield or very high elasticity to be ground into a powder (ESI Fig. 4†). As illustrated in ESI Fig. 5,† the synthesized PIL was easy to be ground into a powder for the subsequent preparation of effervescent tablets, when the amount or volume of anhydrous ethanol and AIBN was 20 mL and 60 mg, respectively, in the presence of 5.0 g [VBTHEA]Cl. As such, the highest yield up to 4.48 g was achieved with a yield rate of 89.6%. Consequently, the above proportion for the amounts of reactive precursors was chosen in the synthesis of P[VBTHEA]Cl.

### 3.3. Optimization of the extraction conditions in the QAPI-EDM procedures

The spiked samples containing five SAs at 20 μg L^−1^ were prepared by fortifying the stock standard solutions into 200 mL deionized water. A series of important variables were optimized, including the amount of P[VBTHEA]Cl, extraction time, type and amount of elution solvent, solution pH, and elution time, were rigorously investigated to obtain high average extraction recoveries (ERs) for five SAs in the water samples.

#### 3.3.1. Amount of P[VBTHEA]Cl

**Fig. 5:**
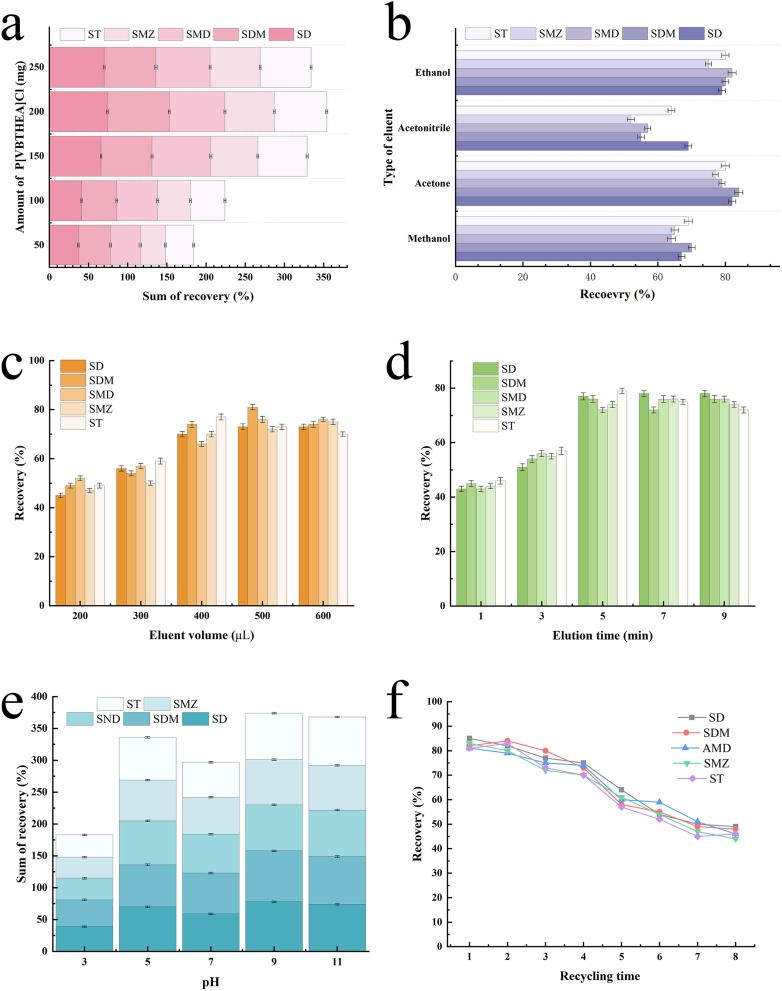
Optimization of the important variables for the QAP-EDSE method. (a) Amount of P[VBTHEA]Cl, (b) type of elution solvent, (c) volume of eluent, (d) solution time, (e) Solution pH, and (f) recyclability of P[VBTHEA]Cl.

In a constant volume of water sample, a very little amount of PIL will lead to insufficient adsorption and extraction, while a very large amount will produce great loss in the following collection and elution processes, both of which substantially decrease the extraction efficiency. Thus, a series of PIL amount (50–250 mg) was added to 10 mL water sample to investigate the effects on ERs for SAs. As depicted in Fig. 5a, the average ER reached as low as 36.5% at the fortified amount of 50 mg, which resulted from the insufficient adsorption due to little PIL. As expected, the ERs for five SAs increased prominently with the rising PIL amount from 50 to 200 mg. However, they showed a declining trend with further increase from 200 to 250 mg, demonstrating that the adsorption reached a plateau at 200 mg. Consequently, 200 mg P[VBTHEA]Cl was deemed as the optimal amount.

#### 3.3.2. Type and amount of the elution solvent

Elution is a crucial step in the QAPI-EDM procedures for the desorption of all analytes from PILs.^32^ Under the conditions of 200 mg PIL, four kinds of elution solvents were investigated in terms of their respective ERs, including methanol, acetonitrile, acetone, and ethyl alcohol. As displayed in Fig. 5b, acetone offered the highest average ER for SAs (∼84.2%), followed by ethyl alcohol (80.1%); however, acetonitrile yielded the lowest elution efficiency (58.4%). Varying water-soluble solvents have differential polarities (log^Kow^ values), thereby leading to “high-to-low” extraction efficiencies for SAs based on the “like-dissolves-like” principle. Building upon the above data, we chose acetone as the appropriate elution solvent in the subsequent trials.

As for the volume of the elution solvent, a very small amount is not enough for the complete elution of analytes from the extractant, while a very large amount causes increased cost and solvent pollution. According to the preliminary experiment results, we investigated the effects of varying volumes (200–600 μL) of acetone on ERs (Fig. 5c). Obviously, the increasing acetone volumes (200–400 μL) led to a gradual increase in the average ERs from ∼48.6% to 72.4%. In contrast, further rise in acetone volumes (400–600 μL) caused slight changes in ERs. Hence, 400 μL was adopted in the subsequent trials.

#### 3.3.3. Effect of elution time

In the effervescence-enhanced extraction process, SAs should be transferred from the sample solution to the sorbent, which is a time-dependent transfer process.^33^ As displayed in Fig. 5d, the elution time was optimized from 1 to 9 min. With the prolongation of elution time from 1 to 5 min, the average ERs for SAs increased monotonically from ∼32.5% to 56.7%, while they remained nearly constant (78.3%) when the elution time varied from 5 to 9 min, implying the achievement of an extraction equilibrium. Therefore, 5 min was selected as the optimal elution time.

#### 3.3.4. Optimization of solution pH

Because the solution pH determines the molecular or ionic state of analytes, it can produce significant influences on the ERs.^33^ In the pH range of 3.0–11.0, the highest average ER (92.5%) for SAs was achieved at pH 9.0 (Fig. 5e). When the solution pH was decreased to 3.0 or 5.0, the extraction efficiency for five SAs by P[VBTHEA]Cl-based effervescent tablet was significantly reduced. Relatively, the average ERs showed an increasing trend at pH 7.0. Boreen *et al.*^34^ reported that SAs could undergo the acid–base process and the cationic (SH^2+^), neutral (SH), and anionic (S^−^) forms are attributed to the protonation and deprotonation of the amino and sulfonamide groups. When the solution pH was higher than p*K*_a,1_ and p*K*_a,2_, the amino and sulfonamide groups exist predominantly (91%) in the neutral and ionized forms, respectively.^34^ The p*K*a values of the five SAs in aqueous solution ranged from 5.0 to 7.0, resulting in the SAs to be positively charged under acidic conditions and negatively charged under alkaline conditions (ESI Table 1†).^35^ Under acidic conditions with pH < 3.7, the main forms of SDM in the solution are SDM^+^ (sulfadimethoxine particles with positive nuclei) and part of SDM^0^ (sulfadimethoxine particles with neutral electricity), and SDM^−^ (sulfadimethoxine particles with negative nuclei) is almost absent (Fig. 4c). When the pH value varies between 3.7 and 6.2, SDM^0^ gradually increases, while SDM^+^ gradually decreases. As the solution is neutral, SDM^+^ almost disappears, and SDM^0^ becomes the main particle in the solution. When the solution pH ranges from 6.2 to 8.6, the number of SDM^0^ decreases gradually, the number of SDM^−^ increases gradually, and there is almost no SDM^+^. Under alkaline conditions with pH value > 8.6, SDM^−^ is the main particle in the solution.^36^ The predominant forms of SDM were anionic state under alkaline conditions, and the surface of PILs P[VBTHEA]Cl was positively charged. Therefore, the phenomenon of positive-negative charge attraction leads to a strong extraction efficiency for SAs. As a consequence, all the subsequent experiments were conducted at a solution pH of 9.0.

### 3.4. Ratio of acidic source to alkaline source in an effervescent tablet

As two of the most important components in effervescent tablets, the molar ratio of acidic source to alkaline source can significantly affect the extraction or adsorption efficiency. If the proportion is not appropriate, it will not only lead to insufficient quantification of CO_2_ bubbles but also trigger strong acidic or alkaline solution pH after an effervescent reaction. In the first case, vigorous dispersion from CO_2_ bubbles cannot be achieved, while in the second case, the inappropriate solution pH influences the percentage of molecular and ionic states of SAs, both of which are not conducive to the highly efficient extraction of analytes. According to our previous report, NaHCO_3_ and TTA were selected as acidic and alkaline sources, respectively. When the proportions of acidic and alkaline sources decreased from 1.50 : 1.00 to 1.00 : 1.50, the produced CO_2_ amount showed an upward trend firstly from 10.30 to 15.67 mg, but a declining trend from 15.67 to 9.77 mg (ESI Table 3†). In addition, the longest and shortest disintegration time was observed to be 174 and 111 s, respectively, at the acidic–basic mass ratio of 1.00 : 1.25 and 1.00 : 1.50. Overall, the investigated acidic–basic ratios did not cause in the significantly differential disintegration time across the range of 2–3 min. Moreover, the solution pH prominently increased with the decreasing acidic–basic ratios from 4.59 to 7.03 under an initial solution of pH 7.0 (ESI Table 3†). Based on a comprehensive consideration of disintegration time and CO_2_ amount produced, we selected the 1.25 : 1.00 acidic–basic mass ratio as the appropriate component of an effervescent tablet.

### 3.5. Reusability of P[VBTHEA]Cl in effervescent tablets

The reusability of PILs is an important indicator for improving the application potential of solidified ILs.^37^ We analyzed the reusability of the P[VBTHEA]Cl adsorbent by regenerating with three washing cycles of ethanol and water, and subsequent drying for use in another effervescent-tablet preparation and extraction cycle. As a result, the as-fabricated effervescent tablets could be reused for at least four cycles with average ER losses of < 11.8% (Fig. 5f), confirming that the as-synthesized PIL retains excellent recyclability and stability. Thus, we posit that P[VBTHEA]Cl is endowed with excellent characteristics for long-term use in the monitoring of trace SAs in environmental waters.

### 3.6. Analytical performance of the QAP-EDSE method

Under the optimized conditions (200 mg of sorbent, 400 μL of eluent, and solution pH 9.0 in 8 mL water sample), the analytical performance of the QAP-EDSE method was evaluated based on the following metrics: linear ranges (LRs), coefficient of determination (*R*^2^), limits of detection (LODs), limits of quantification (LOQs), as well as intraday and interday precision. As generalized in Table 1, the LRs spanned the range of 1.0–500 μg L^−1^ for ST, SMD, SDM, SMZ, and 0.5–500 μg L^−1^ for SD, with all *R*^2^ values > 0.9976. Based on the signal to noise of 3 and 10 (S/N = 3, 10), the LODs and LOQs for five SAs were 0.11–0.31 μg L^−1^ and 0.36–1.03 μg L^−1^, respectively. At three spiking levels (5 μg L^−1^ (low), 20 μg L^−1^ (middle), and 50 (high) μg L^−1^), the interday and intraday precision, expressed as relative standard deviations (RSDs), were in the range of 1.9–5.1% and 2.7–6.7%, respectively. Collectively, these performance metrices evidence that the QAP-EDSE method can satisfy the technical requirements for the trace-level detection of SAs in environmental waters.

**Table 1:** Analytical performance of the QA-EDSE/LC-DAD method in environmental waters^a^

Analytes	LR (μg L^−1^)	*R* ^2^	LODs (μg L^−1^)	LOQs (μg L^−1^)	Intraday precision (RSD%, *n* = 6)			Interday precision (RSD%, *n* = 6)		
					Low	Medium	High	Low	Medium	High
ST	1.0–500	0.9976	0.21	0.70	4.2	3.4	2.7	6.7	4.4	3.3
SD	0.5–500	0.9994	0.11	0.36	4.8	4.1	2.2	6.3	4.9	2.7
SMD	1.0–500	0.9989	0.19	0.64	5.1	3.6	1.9	5.4	5.2	3.1
SDM	1.0–500	0.9985	0.31	1.03	4.6	4.1	2.2	6.3	4.3	3.7
SMZ	1.0–500	0.9992	0.22	0.73	4.6	3.5	3.1	5.3	5.3	3.1

a Notes: LR, *R*^2^, LODs, and LOQs denote the abbreviations of linear range, coefficient of determination, limits of detection, and limits of quantification, respectively.

### 3.7. Analyses of SAs by the QAP-EDSE/LC-DAD method in real-world waters

**Fig. 6:**
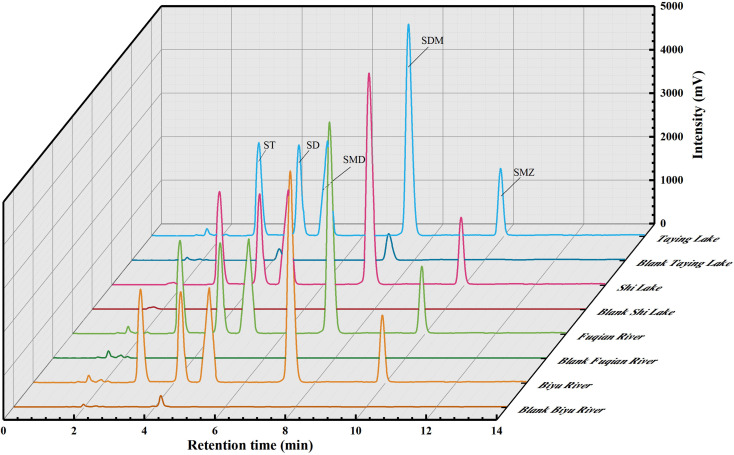
Typical chromatograms of SAs in real-world water samples by the QAP-EDSE/LC-DAD method.

The chromatographic profiles for the standards of SAs are elaborated in Fig. 4d, and the retention times of ST, SD, SMD, SDM, and SME were 3.05, 4.19, 5.00, 7.29, and 9.91 min, respectively. Typical chromatograms for five SAs are shown in Fig. 6 in the unspiked (blank) and spiked (20 μg L^−1^) samples, including the Taying lake, Shi lake, Fuquan river, and Biyu river waters.

In addition, the total ion chromatograms with very good separation of 5 kinds of SAs in the spiked (20 μg L^−1^) Taying lake sample are shown in ESI Fig. 6†, and the retention times of SD, ST, SMD, SMZ, and SDM were 3.84, 4.05, 5.02, 5.68, and 6.83 min, respectively. By the present QAP-EDSE/LC-DAD method, SD was detected to be 0.61 and 0.94 μg L^−1^, respectively, in the Biyu river and Taying lake, respectively, while SDM was monitored to be 1.50 μg L^−1^ in Taying lake (Table 2). When three concentrations of SAs (5, 20, and 50 μg L^−1^) were fortified in the blank water samples, the relative recoveries for SAs spanned the range of 81.40–102.62%. These data document that the newly developed method is feasible for SAs detection with high accuracy and precision in real-world water samples.

**Table 2:** The fortified recoveries for SAs in real-world water samples

Analyte	Blank	Added (μg L−1)	Detected (μg L−1)	RR (%)	Added (μg L−1)	Detected (μg L−1)	RR (%)	Added (μg L−1)	Detected (μg L−1)	RR (%)
ST	ND	5	4.82 ± 0.21	96.44	20	19.42 ± 0.63	97.10	50	47.12 ± 0.99	94.24
SD	0.61		5.08 ± 0.17	89.40		19.84 ± 0.61	96.15		49.85 ± 0.91	98.48
SMD	ND		4.64 ± 0.18	92.80		18.58 ± 0.71	92.90		43.65 ± 0.95	87.30
SDM	ND		4.38 ± 0.11	87.60		17.42 ± 0.54	87.10		48.65 ± 0.11	97.30
SMZ	ND		4.88 ± 0.18	97.60		18.45 ± 0.52	92.25		45.43 ± 0.78	90.86
ST	ND	5	4.75 ± 0.16	95.00	20	19.51 ± 0.64	97.55	50	49.22 ± 0.82	98.44
SD	ND		4.87 ± 0.17	97.40		19.94 ± 0.71	99.70		49.45 ± 0.93	98.90
SMD	ND		4.38 ± 0.19	87.60		19.95 ± 0.63	99.75		43.62 ± 0.65	87.24
SDM	ND		4.73 ± 0.17	94.60		18.66 ± 0.52	93.30		46.61 ± 0.78	93.22
SMZ	ND		4.81 ± 0.17	96.20		17.94 ± 0.55	89.70		49.23 ± 0.79	98.46
ST	ND	5	4.52 ± 0.07	90.40	20	17.41 ± 0.62	87.05	50	47.26 ± 0.94	94.52
SD	ND		4.94 ± 0.13	98.80		19.34 ± 0.61	96.70		46.33 ± 0.87	92.66
SMD	ND		4.93 ± 0.07	98.60		17.47 ± 0.52	87.35		48.64 ± 0.82	97.28
SDM	ND		4.81 ± 0.09	96.20		18.13 ± 0.49	90.65		49.32 ± 0.74	98.64
SMZ	ND		4.94 ± 0.11	98.80		19.14 ± 0.61	95.70		44.71 ± 0.77	89.42
ST	ND	5	4.34 ± 0.13	86.80	20	17.67 ± 0.53	88.35	50	44.21 ± 0.85	88.42
SD	0.94		5.01 ± 0.15	81.40		19.25 ± 0.52	91.55		49.16 ± 0.87	96.44
SMD	ND		4.63 ± 0.16	92.60		17.44 ± 0.62	87.20		44.73 ± 0.79	89.46
SDM	1.5		5.57 ± 0.19	81.40		19.42 ± 0.58	89.60		52.81 ± 0.85	102.62
SMZ	ND		4.82 ± 0.17	96.40		18.35 ± 0.61	91.75		49.83 ± 0.77	99.66

### 3.8. Comparison of the QAP-EDSE/LC-DAD with previous methods

The newly developed method was compared with the previously reported methods in the context of analytical indexes such as ERs, RSDs, LODs, extraction time, and amount of sorbent (Table 3). The effervescence tablets used in this QAP-EDSE method can be prepared in advance and stored in a desiccator until required without any deterioration, and the analytical operations are very simple. Many approaches have been reported for the assay of SAs, such as SPE,^38^ SPME,^39^ magnetic solid-phase extraction (MSPE),^40^ molecularly imprinted polymer solid-phase extraction (MIP-SPE),^41^ and oxidized buckypaper for stir-disc solid phase extraction (BP@SD-SPE).^42^

**Table 3:** Comparison of the QAP-EDSE/LC-DAD method with previously reported methods for SAs detection in waters^a^

Pretreatment methods	Samples	SAs	Amount of sorbent (mg)	Pretreatment time (min)	RSD (%)	LOD (mg L^−1^)	Recovery (%)	Ref
SPE/LC-UV	Seawater	SAA, SDZ, SRZ, SCT, STZ, SDX, SMX, SMZ	540	5	1.2–6.4	0.19–0.50	99–104	38
SPME/LC-UV	Wastewater	SP, SMZ, SIZ, SM2, SDM	60	40	0.29	0.30–0.35	87	39
MSPE/LC-UV	Environmental water	SPD, SMR, SME, SMM, SCP, SD	50	8	9.8–10.7	0.09–0.16	74.2–104.1	40
MIP-SPE/LC-MS	Environmental water	SMX, SMM, SMD, SDM, SQX	20	15	3.6–7.2	0.003–0.0047	62.2–91.1	41
BP@SD-SPE	Surface water	SA, SMZ, SMR, SDZ, SMT, SCP, SDX, SQX, SG	908 mm^2^ per side	1440 (24 h)	15	0.003–0.286	3.0–49	42
QAP-EDME/LC-DAD	Environmental water	ST, SD, SMD, SDM, SMZ	200	< 5	1.9–6.7	0.11–0.31	81.40–102.62	This work

a Notes: (1) SPE: solid-phase extraction; (2) SPME: solid-phase microextraction; (3) MSPE: magnetic solid-phase extraction; (4) MIP-SPE: molecularly imprinted polymer solid-phase extraction; (5) BP@SD-SPE: oxidized buckypaper for stir-disc solid phase extraction; (6) QAP-EDME: quaternary ammonium poly ionic liquids effervescence-enhanced dispersive solid-phase extraction.

The recoveries (81.40–102.62%) by this proposed method is comparable with other pretreatment methods and better than MIP-SPE (62.2–91.1%)^41^ and BP@SD-SPE (3.0–49%).^42^ The LODs (0.11–0.31 μg L^−1^) offered by the newly developed are substantially lower than SPE (0.19–0.50 μg L^−1^)^39^ and SPME (0.30–0.35 μg L^−1^),^39^ but higher than MSPE (0.09–0.16 μg L^−1^)^41^ combined with MS detector (MIPs-SPE and BP@SD-SPE).^41,42^ The RSDs are in the range of 1.9–6.7%, which are comparable with SPE (1.2–6.4%)^38^ and MIP-SPE (3.6–7.2%),^41^ and much better than that of MSPE (9.8–10.7%)^40^ and BP@SD-SPE (15%).^42^ The amount of adsorbent (200 mg of PIL) is much lower than SPE (500 mg).^38^ But it is worth noting that the sample preparation time (<3 min) by the present method is in general agreement with that by SPE (5 min),^38^ and much shorter than most other pretreatment methods, such as 40 min for SPME, 8 min for MSPE,^40^ 15 min for MIP-SPE,^41^ and over 24 h for BP@SD-SPE.^42^ Compared to the previous methods mentioned above, this proposed method does not require the use of organic solvents to activate and elute the solid-phase extraction column, which effectively reduces the volume of organic solvents. With the aid of high extraction efficiency of P[VBTHEA]Cl to SAs and excellent dispersion of effervescent reaction, it can quickly complete the extraction of target SAs within 5 min. Therefore, the QAP-EDSE method can quickly and efficiently analyze and detect trace SAs in environmental waters.

## 4. Conclusions

Herein, a PIL P[VBTHEA]Cl was synthesized and characterized by a series of spectral techniques. The solid P[VBTHEA]Cl overcame the drawbacks of conventional ILs, such as high viscosity, difficult sampling and retrieval, as well as easy volume loss in aqueous solution, when used in the extraction or microextraction procedures. This PIL was employed as an extraction sorbent and pressed into an effervescent tablet for developing a fast and “green” QAP-EDSE approach. The tablet precursors were composed of P[VBTHEA]Cl, TTA, NaHCO_3_, and water-soluble starch, which could be quickly dispersed by a vigorous effervescent reaction from acidic and alkaline sources. The prominent advantages of the QAP-EDSE method lie in that it realizes the integration of rapid enrichment, extraction, and dispersion into one synchronous step with the aid of vigorous CO_2_ bubbles. Based on a series of satisfactory performance metrics for SAs detection, we posit that the QAP-EDSE/LC-DAD method is a promising pretreatment alternative as it offers good accuracy and reproducibility without use of any special instruments.

## Conflicts of interest

The authors declare no conflict of interest.

## Supplementary Material

RA-012-D2RA02488H-s001
