# Supplementary material for: Trace-level detection of sulfonamide antibiotics using quaternary ammonium polymeric ionic liquid-based effervescence-enhanced dispersive solid-phase extraction followed by LC-DAD analysis in environmental waters
Source: RSC Adv. 2022 Oct 19;12(46):29915–27. doi: 10.1039/d2ra02488h (PMC9580476; doi:10.1039/d2ra02488h)
Supplement: RA-012-D2RA02488H-s001 [file RA-012-D2RA02488H-s001.pdf]

Electronic Supplementary Material (ESI) for RSC Advances.
This journal is © The Royal Society of Chemistry 2022

Supplementary Figure captions:

Supplementary Fig. 1. The schematic diagram of the synthetic routes for [VBTHEA]Cl (a) and P[VBTHEA]Cl (b).

Supplementary Fig. 2. The photographs of the synthetic [VBTHEA]Cl and P[VBTHEA]Cl. (a) [VBTHEA]Cl when fortified at the ethanol volume of 30 mL, (b) [VBTHEA]Cl when fortified at the ethanol volume of 20 mL, and (c) [VBTHEA]Cl when fortified at the ethanol volume of 10 mL. (d) P[VBTHEA]Cl when fortified at the ethanol volume of 30 mL, (e) P[VBTHEA]Cl when fortified at the ethanol volume of 20 mL, and (f) P[VBTHEA]Cl when fortified at the ethanol volume of 10 mL.

Supplementary Fig. 3. The profiles for each step in the QAP-EDSE procedures

Note: (a) addition of 8 mL sample solution; (b) addition of effervescent tablets; (c) the generation of CO_2; (d) homogenous solution after effervescence; (e) absorbents settle after the end of effervescence reaction; (e) solid-liquid phase separation after station.

Supplementary Fig. 4. The photographs of the synthetic P[VBTHEA]Cl. (a) The fortified dosage of AIBN at 50 mg, (b) 60 mg, and (c) 70 mg.

Supplementary Fig. 5. Optimization of the synthetic conditions. (a) Effect of the ethanol volume on the yield of P[VBTHEA]Cl; and (b) Effect of the fortified amount of AIBN on the yield of P[VBTHEA]Cl.

Supplementary Fig. 6. Total ion chromatograms of five kinds of sulfonamides in real-world water sample (20 μg L^{-1})

Supplementary Table legends:

Supplementary Table 1. The physico-chemical properties and molecular structures of five SAs

Supplementary Table 2. Mass spectrometric parameters for five SAs

Supplementary Table 3. Effects of varying acid-base mass ratios on the physico-chemical properties of effervescent tablets

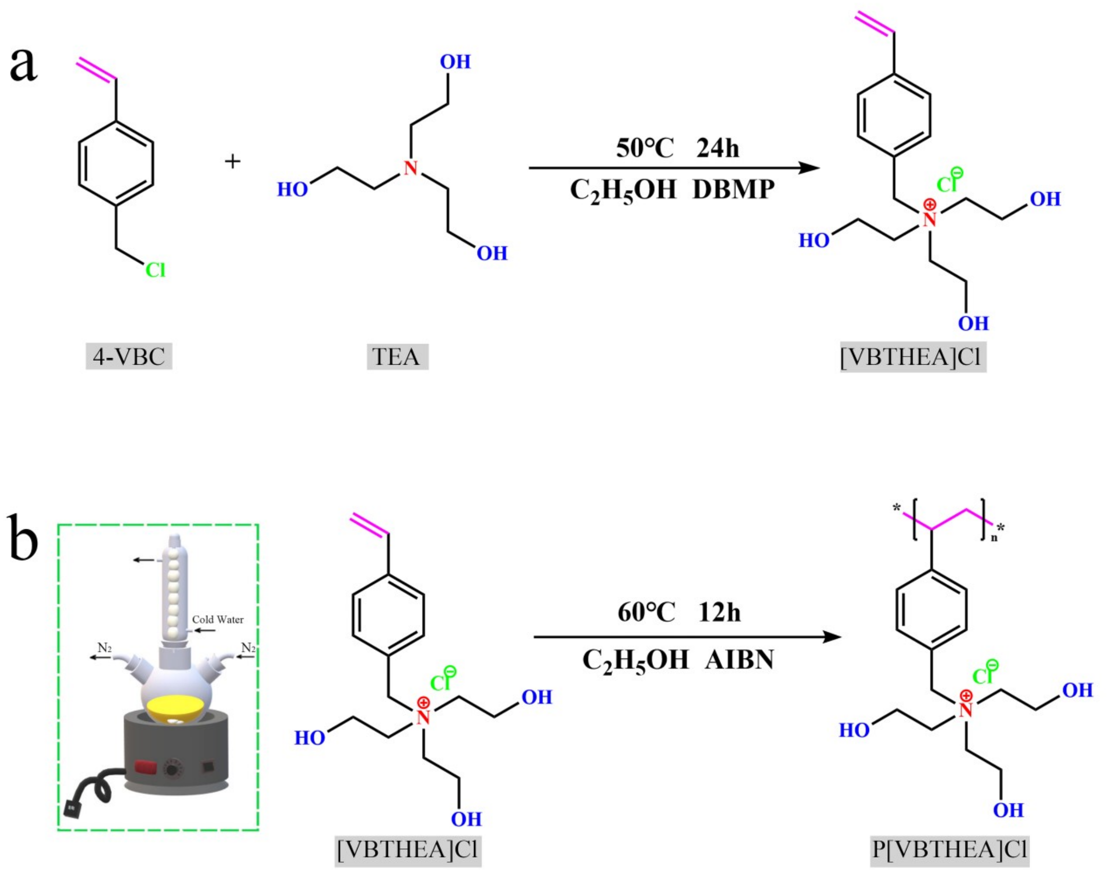

Supplementary Fig. 1

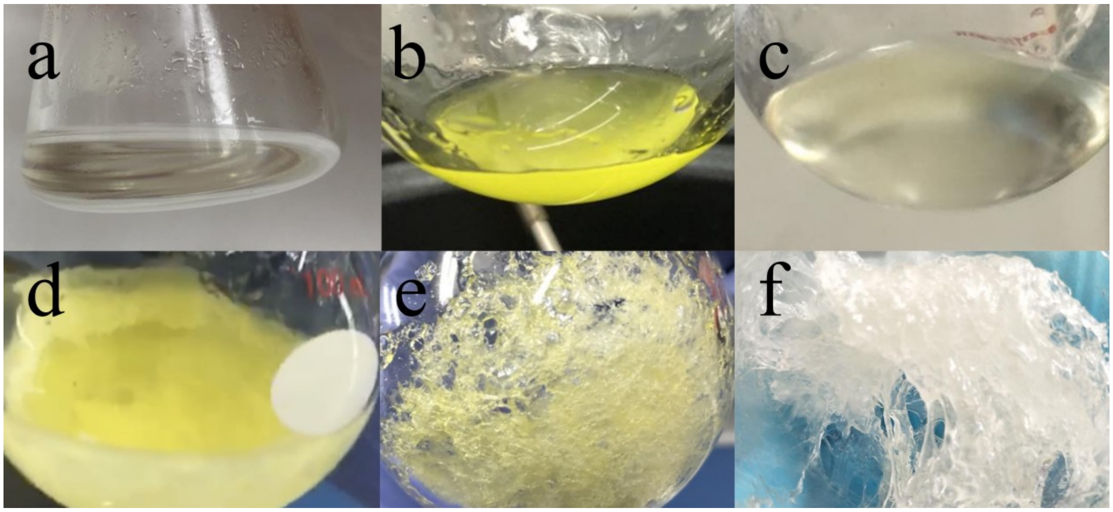

Supplementary Fig. 2

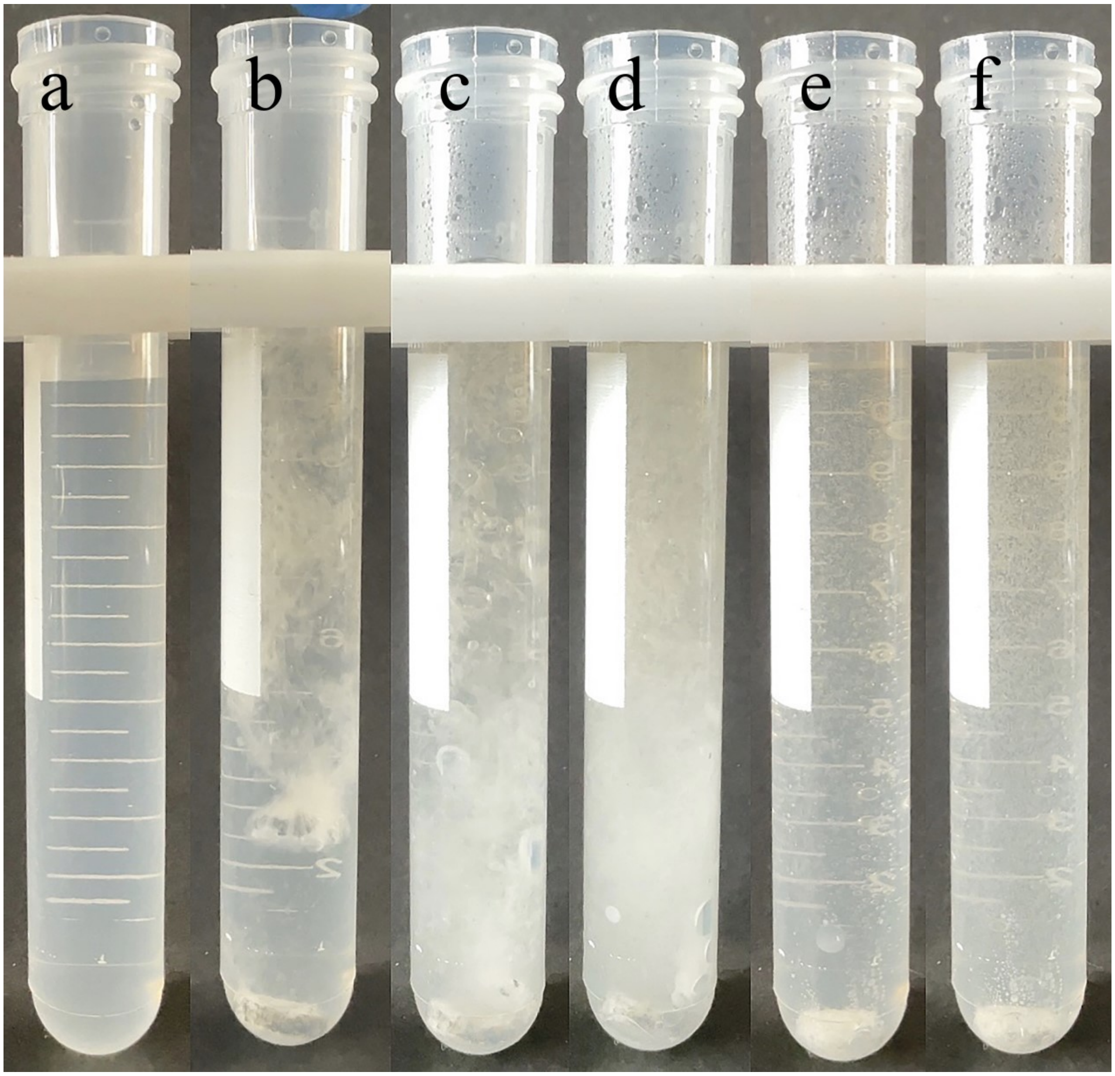

Supplementary Fig. 3

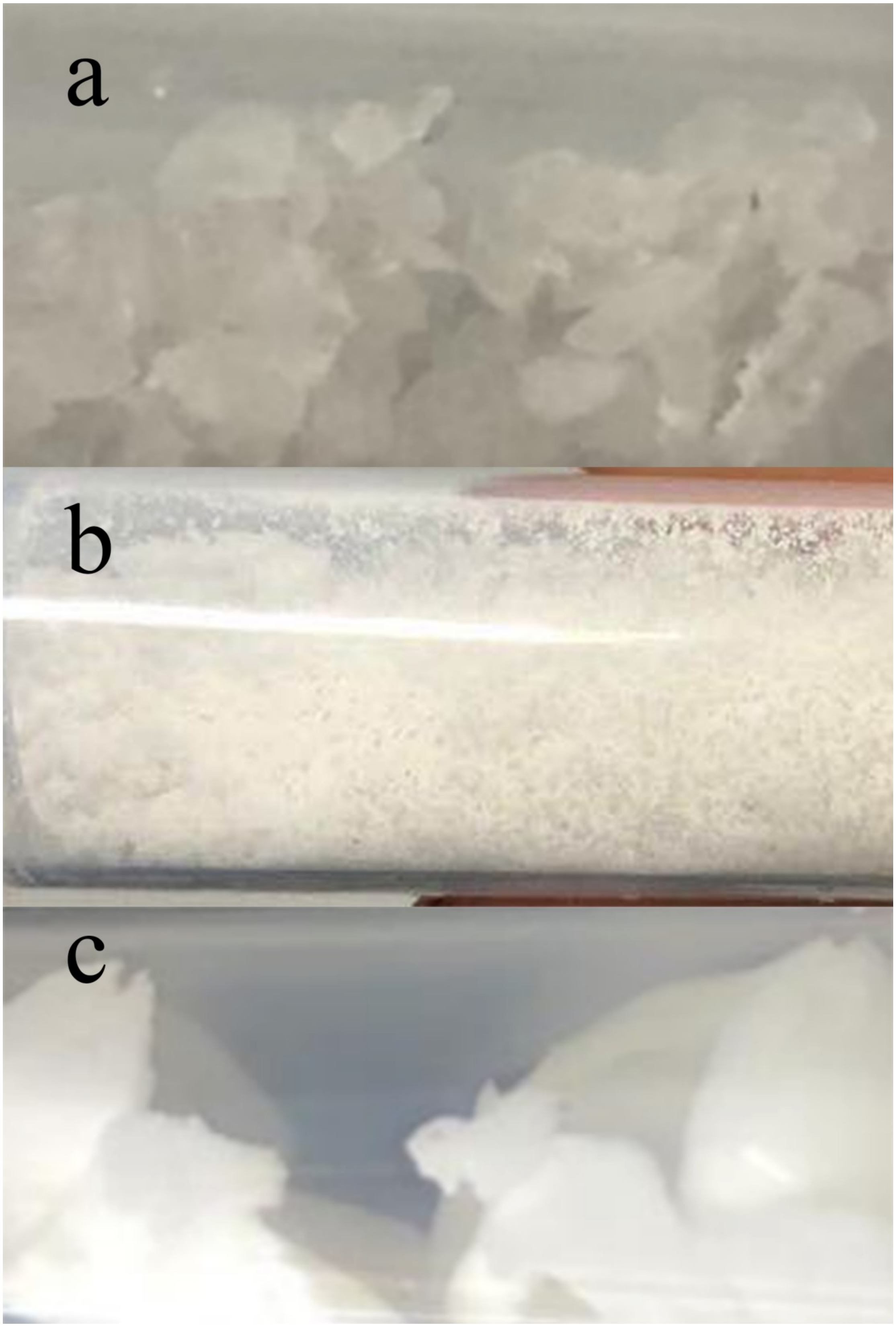

Supplementary Fig. 4

a

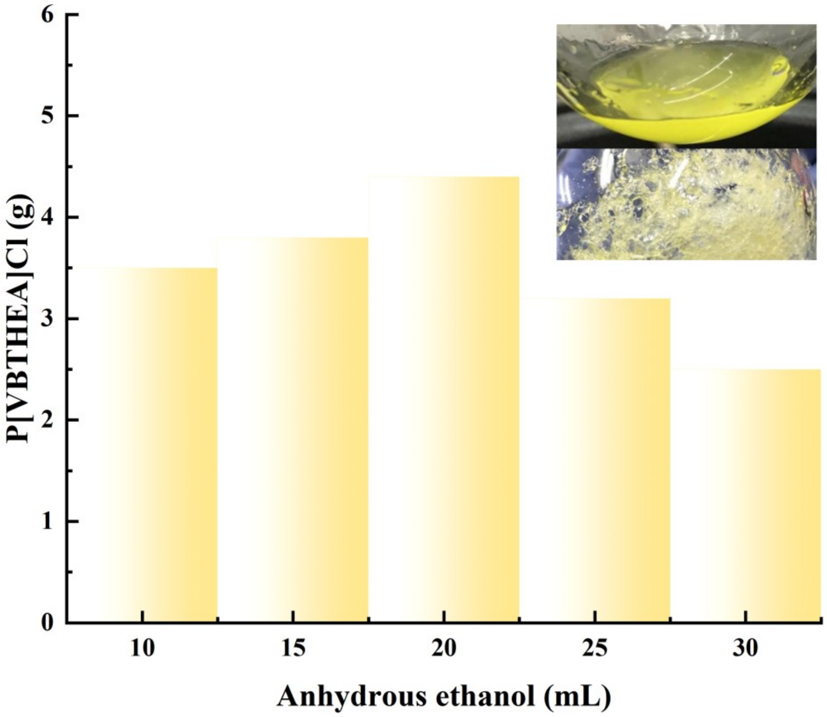

P[VBTHEA]Cl (g)
0
1
2
3
4
5
6
10
15
20
25
30
Anhydrous ethanol (mL)

b

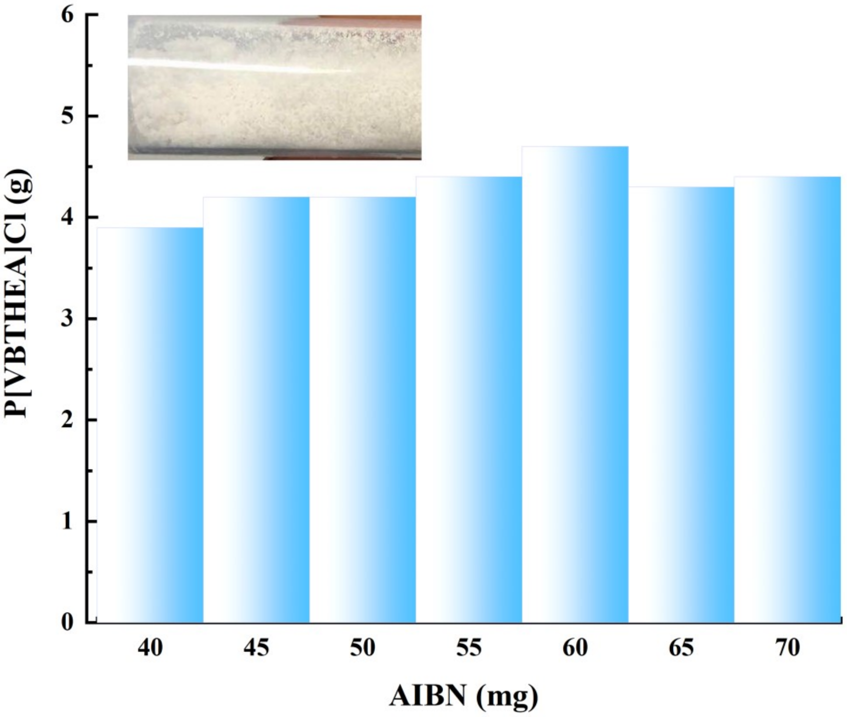

P[VBTHEA]Cl (g)
0
1
2
3
4
5
6
40
45
50
55
60
65
70
AIBN (mg)

Supplementary Fig. 5

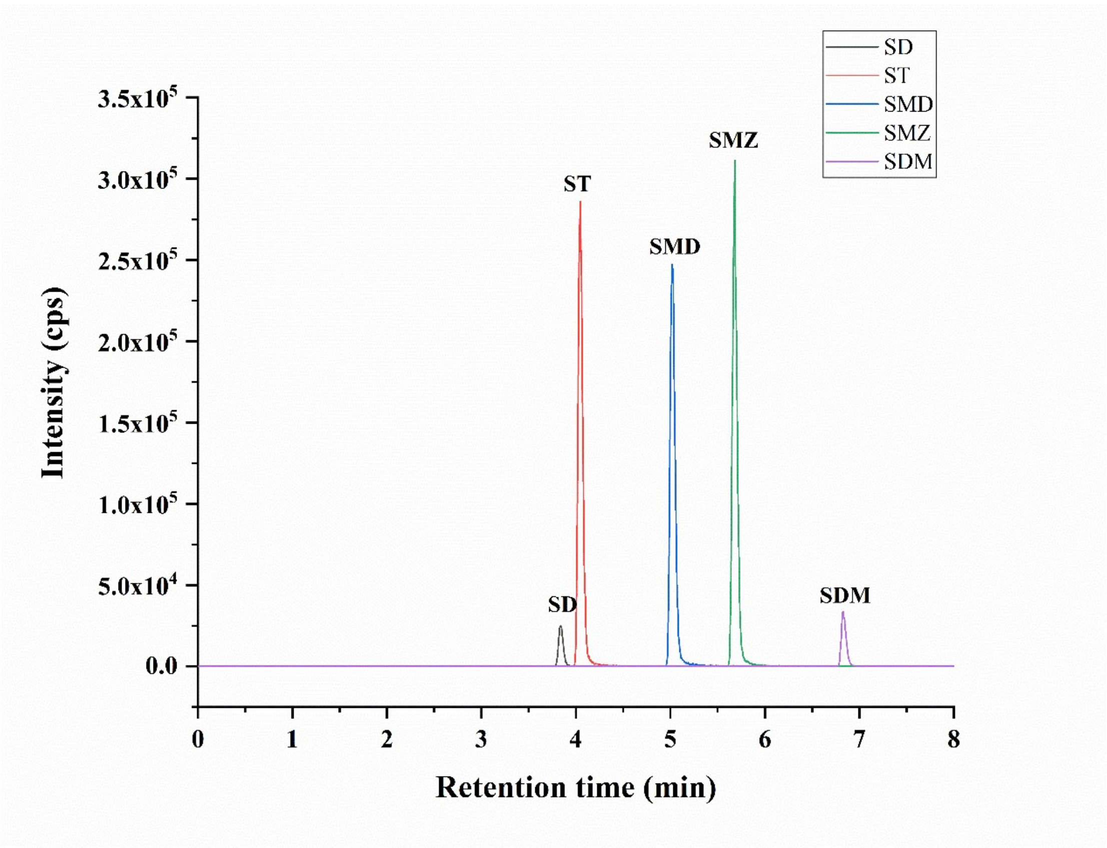

Supplementary Fig. 6

Supplementary Table 1. The physico-chemical properties and molecular structures of five SAs

Compounds	CAS No.	M.W.	p$K_{a,1}$	p$K_{a,2}$	log^{Kow}	Molecular structure
Sulfamethoxydiazine (SMD)	651-06-9	280.3	7.02	-	0.25	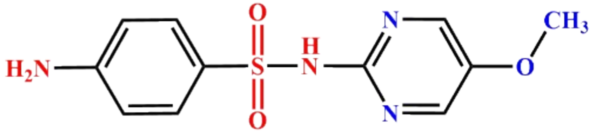
Sulfadimethoxine (SDM)	122-11-2	310.33	6.2	-	0.78	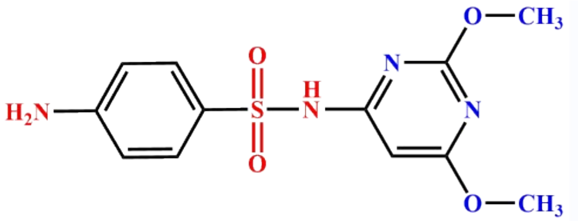
Sulfathiazole (ST)	72-14-0	255.32	2.2	7.2	0.05	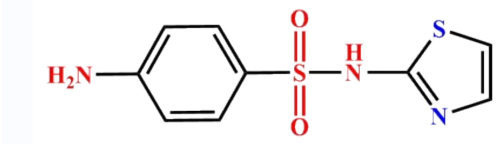
Sulfamethoxazole (SMZ)	723-46-6	253.28	1.6	5.7	0.89	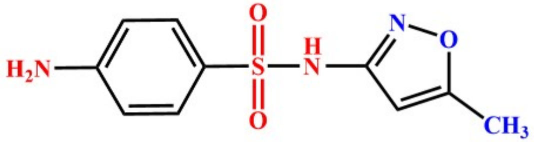
Sulfadiazine (SD)	68-35-9	250.28	2.21	6.4	0.05	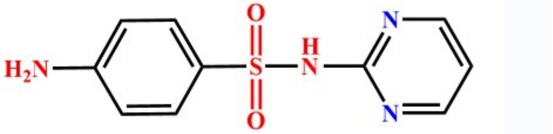

Supplementary Table 2. Mass spectrometric parameters for five SAs

Compounds	Ionization mode	Parent ion (m/z)	Product ion (m/z)	Collision energy (eV)	Cone voltage (V)
SD	ESI^+	251.2	156.2/108.2	56	30
ST	ESI^+	256.2	156.1/108.2	58	33
SMD	ESI^+	281.1	156.2/108.2	64	36
SMZ	ESI^+	254.2	156.1/108.2	63	31
SDM	ESI^+	311.1	156.1/108.1	69	36

Supplementary Table 3. Effects of varying acid-base mass ratios on the physico-chemical properties of effervescent tablets

Acid-base ratio/(g:g)	Disintegration time (s)	Solution pH after reaction	CO_2 amount (mg)
1.50:1.00	131	4.59	10.30
1.25:1.00	137	5.19	15.67
1.00:1.00	153	6.63	12.62
1.00:1.25	174	6.90	12.51
1.00:1.50	111	7.03	9.77
